# Arsenic Exposure and Age- and Sex-Specific Risk for Skin Lesions: A Population-Based Case–Referent Study in Bangladesh

**DOI:** 10.1289/ehp.9207

**Published:** 2006-08-24

**Authors:** Mahfuzar Rahman, Marie Vahter, Nazmul Sohel, Muhammad Yunus, Mohammad Abdul Wahed, Peter Kim Streatfield, Eva-Charlotte Ekström, Lars Åke Persson

**Affiliations:** 1 ICDDR, B: Centre for Health and Population Research, Mohakha, Dhaka, Bangladesh; 2 Institute of Environmental Medicine, Karolinska Institutet, Stockholm, Sweden; 3 International Maternal and Child Health (IMCH), Uppsala University, Sweden

**Keywords:** arsenic skin lesions, Bangladesh, case–referent study, dose response, drinking water, exposure, sex

## Abstract

**Background:**

The objective of this population-based case–referent study in Matlab, Bangladesh, was to assess the susceptibility to arsenic-induced skin lesions by age and sex, in a population drinking water from As-contaminated tube wells.

**Methods:**

Identification of As-related skin lesions was carried out in three steps: *a*) screening of the entire population > 4 years of age (*n* = 166,934) by trained field teams; *b*) diagnosis of suspected As-related cases by physicians; and *c*) confirmation by experts based on physicians’ records and photographs. A total of 504 cases with skin lesions were confirmed. We randomly selected 2,201 referents from the Matlab health and demographic surveillance system; 1,955 were eligible, and 1,830 (94%) were available for participation in the study. Individual history of As exposure was based on information obtained during interviews and included all drinking-water sources used since 1970 and concentrations of As (assessed by atomic absorption spectrophotometry) in all the tube wells used.

**Results:**

Cases had been exposed to As more than referents (average exposure since 1970: male cases, 200 μg/L; female cases, 211 μg/L; male referents, 143 μg/L; female referents, 155 μg/L). We found a dose–response relationship for both sexes (*p* < 0.001) and increased risk with increasing socioeconomic status. Males had a higher risk of obtaining skin lesions than females (odds ratio 10.9 vs. 5.78) in the highest average exposure quintile (*p* = 0.005). Start of As exposure (cumulative exposure) before 1 year of age was not associated with higher risk of obtaining skin lesions compared to start of As exposure later in life.

**Conclusions:**

The results demonstrate that males are more susceptible than females to develop skin lesions when exposed to As in water from tube wells.

The discovery of arsenic in drinking water in many areas of the world has caused widespread public health concern. Close to 100 million people in the world, including about 13 million in the United States, are chronically exposed to inorganic As [International Agency for Research on Cancer (IARC) 2004]. The As problem in Bangladesh is perhaps the most devastating, because about half of the total 6–11 million hand-pumped tube wells yield drinking water with As concentrations > 10 μg/L, the drinking-water guideline recommended by the World Health Organization (WHO) [British Geological Survey (BGS) 2001; Smith et al. 2000].

Inorganic As is an established potent human carcinogen (IARC 2004). In addition, ingestion of As through drinking water has been implicated in several noncancer diseases, for example, peripheral vascular disease; hypertension; respiratory, neurologic, and liver disorders; and diabetes mellitus [IARC 2004; National Research Council (NRC) 2001; WHO/IPCS (International Programme on Chemical Safety) 2001]. Early effects of exposure to As in drinking water include pigmentation changes and hyperkeratosis (IARC 2004; Smith et al. 2000), which reportedly appear after 5–10 years of exposure (Guha Mazumder et al. 1998). These skin lesions may develop into more serious and disabling forms, including cancer (Guha Mazumder et al. 1998; Haque et al. 2003; IARC 2004; NRC 2001; Tondel et al. 1999; Tseng 1977; WHO/IPCS 2001). Because of the magnitude of the problem and the difficulties involved in mitigation (Jakariya et al. 2005), it is essential to identify risk groups in the population (NRC 2001). Numerous studies on As-related health effects have been performed, particularly in recent years. Still, few have focused on susceptibility factors.

Our ongoing studies on As-induced health effects in Matlab, Bangladesh, showed that the highest prevalence of As-induced skin lesions occurred in middle-aged men (Rahman et al. 2006), suggesting variation in susceptibility by sex and age. A few previous reports have indicated that men are more affected by As-related skin effects, including skin cancer, than women (Chen et al. 2003; Ferreccio et al. 2000; Guha Mazumder et al. 1998; Kadono et al. 2002; Tseng 1977; Watanabe et al. 2001), whereas other studies found women to be more susceptible than men (Ahmad et al. 1999) or did not identify any difference (Ahsan et al. 2000; Hadi and Parveen 2004; Tondel et al. 1999). However, none of these studies was designed to study differences between the sexes.

The present population-based case–referent study aims at determining the sex-specific risk of As-induced skin lesions in Matlab, Bangladesh, an area with high prevalence of elevated concentrations of As in tube-well water (Rahman et al. 2006). Further, it aims at assessing whether a start of exposure before 1 year of age compared with later periods is associated with a higher risk of developing the disease. We took advantage of the comprehensive ICDDR, B Health and Demographic Surveillance System (HDSS) in Matlab. By defining the study base as all people > 4 years of age who lived in the demographic surveillance area, the design allowed a novel approach to assess lifetime As exposure and evaluate sex- and age-related differences in risk of obtaining As-related skin effects.

## Methods

### Study base

The study was carried out in Matlab, located 53 km southeast of Dhaka, where the Meghna River joins the confluent streams of the Brahmaputra and Ganges rivers and the bedrock is highly affected by the long-term sedimentation process of As-laden soil from the mountains. The study base was established using the HDSS, covering 142 villages in Matlab, encompassing a population of 220,000 on 18,386 hectares of land. The HDSS recorded all vital events, as well as in- and out-migration since 1963. Births, deaths, marriages, pregnancies, and pregnancy outcomes are registered and updated by community health workers who visit all homes on a monthly basis. The study base consisted of all people > 4 years of age who had lived and consumed drinking water in the HDSS area for at least 6 months prior to the study. Thus, residents of Matlab who had spent most of their time outside the area were not included. In total, 180,811 individuals were eligible for the study, and all were visited in their homes during January 2002–August 2003 (Rahman et al. 2006). In spite of repeated attempts, 13,877 individuals could not be interviewed and examined; therefore 166,934 individuals participated in the study.

All households and individuals were informed about the study and gave written consent to participate. After the interviews and screening for skin lesions had been completed in an area, the As concentrations of the tube wells were tested using a field kit. Tube wells with water containing > 50 μg/L were painted red, and others were painted green (Jakariya et al 2005). The ICDDR, B research review committee and ethical review committee approved the study.

### Case ascertainment

**Figure 1:**
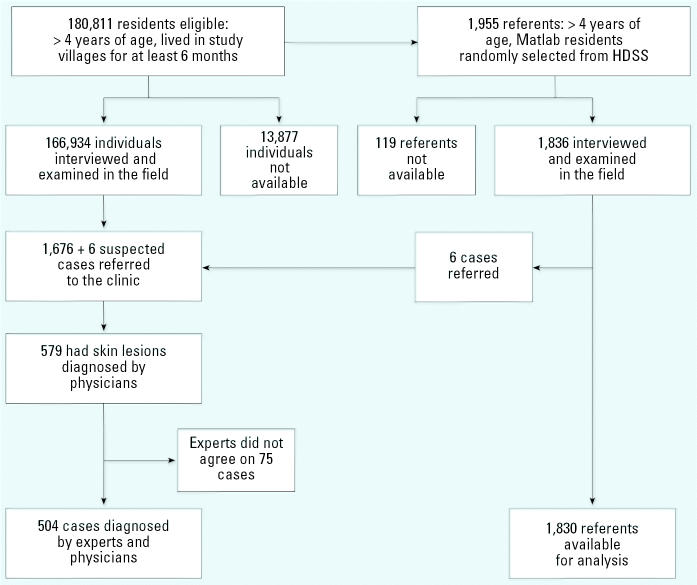
Flow chart showing case identification and selection of referents.

We applied a stepwise procedure for the identification of As-related skin lesions (Figure 1). First, all 166,934 individuals were carefully examined in the field for As-associated skin lesions according to a structured protocol, after extensive training of the field staff (Rahman et al. 2006). Twenty-four field teams, each consisting of one man and one woman, moved from village to village and house to house examining the skin of all participants for skin lesions and interviewing them regarding lifetime water-consumption history. Suspected cases of As-related skin lesions (*n* = 1,682) were referred to study physicians at the health centers for further examination. Physicians diagnosed As-related skin lesions in 579 individuals. They documented (noting the location and appearance of lesions) and photographed all visible or palpable dermal lesions that they considered As-related. Later, two dermatologists independently verified the As-induced skin lesions by inspection of photographs with accompanying documentation. If they disagreed with the physicians’ judgment, the experts and physicians reexamined the patient physically. Experts confirmed 504 of the cases that had been clinically diagnosed. Experts, as well as physicians and field staff, were blinded to the As exposure of the participants.

As-induced skin lesions were classified as follows. Hyperpigmentation (melanosis) usually consists of diffuse dark-brown or blackish areas on the skin of the neck, trunk, or extremities and/or mucous membrane (gum, tongue, buccal mucosa), and diffuse or spotted dense pigmentation on the trunk and other parts of the body. Hypopigmentation (leukomelanosis) is characterized by whitish or pallor patches commonly referred to as raindrop pigmentation. Keratosis is characterized as bilateral thickening of the palms and soles, small protrusions on palms and soles, and occasionally on the dorsum of the hands and feet or as nodulation on limbs.

### Selection of referents

Using the HDSS database, we randomly selected two referents per expected case, a total of 2,201 individuals, from the entire population > 4 years of age. We did not match for age and/or sex to allow analysis of effect modification by these factors. Individuals who did not live in Matlab or drink water from any source in Matlab at least once per week were excluded. Of the 1,955 eligible referents, 119 (6%) were not available during field interviews (nonparticipating referents). In total, 1,836 referents were interviewed and examined in the field. Six persons among the referents were identified with skin lesions and were referred for diagnosis and verification. All were confirmed as As-related cases.

### Exposure assessment

The field teams interviewed all individuals regarding their water-consumption history and recorded the water sources used, including location, during each calendar year since 1970 (or birth, if later than 1970). We chose 1970 as the starting point because the databases did not allow reliable tracking of people’s residence before that year. Also, there were reportedly few tube wells before that date, implying use of surface water with very low As concentrations (WHO/IPCS 2001). We also asked which year individuals started using tube-well water as drinking water because that was an event most people would remember. The reported information on drinking-water sources was validated using results from the household economic surveys conducted in 1974, 1982, and 1996 conducted in Matlab, which contained information on sources of drinking water (Razzaque et al. 1998; Ruzicka and Chowdhury 1978). These social–economic surveys covered the entire population of the Matlab surveillance area and included individual level (demographic data, education, occupation, women’s status) and household-level information (possessions of household items, land, latrine, and source of drinking water as either tube-well or surface water). The information on water sources excerpted from those surveys was printed on the questionnaires and used for instant cross-checking of the responses obtained in the interviews.

The As exposure history was calculated for each participant based on the different water sources used since 1970 and the water-As concentrations of these. Water samples from all functioning tube wells were measured for As concentration by hydride-generation atomic absorption spectrometry (Wahed et al. 2006), whereas surface water (water from ponds, rivers, and collected rain) was assigned a concentration of 0 μg As/L. Many previously used tube wells were found to be nonfunctioning (*n* = 1,946). To reconstruct the historical exposure, we used the average tube-well As concentration of the village as a proxy for nonfunctioning tube wells. That was superior to village median or *bari* (extended household) mean/median as proxy, when we simulated missing data for a number of randomly selected tube wells with known As concentrations. For individuals who had migrated to the study area and previously consumed tube-well water outside the study area, the As concentration was imputed (*n* = 474), using data from the BGS (2001) for the relevant district (mean concentration of the district). Both the average and the cumulative historical As exposure were calculated for each subject. Average As exposure was calculated as the time-weighted mean As concentration of drinking water of all sources used since 1970 or birth. The cumulative As exposure was calculated by summing up the As concentration multiplied by the number of years of usage (μg/L × years) for all water sources used since 1970.

### Potential confounders

In addition to the primary exposure variable, we evaluated other background variables available in the vital records and suspected to be associated with As exposure and the primary outcome, including age, sex, education, and assets score. Data on socioeconomic status (SES) of individuals and households were obtained from two sources: interviews with cases and referents and by linkage to SES data in the HDSS databases. The HDSS databases are repeatedly updated on information regarding education, occupation, and assets. Household economic status was defined by constructing a wealth index using asset ownership. Resulting asset scores were categorized into five groups ranging from one (poorest) to five (richest), based on a model described by Gwatkin et al. (2000). Asset scores were based on a household-level SES census in 1996. Some cases (*n* = 40) and referents (*n* = 188) did not have asset scores because they were absent during the census in 1996, had split households, or had in-migrated after 1996.

### Statistical methods

We compared age, sex, asset score, education, and average lifetime As exposure between the study population and selected referents, and between participating and nonparticipating referents, supported by chi-square testing. We analyzed average As lifetime exposure, cumulative As exposure, and age at first exposure for cases and referents, separately for each sex and supported by analysis of variance.

Age, sex, education, and household asset scores were also compared between cases and referents to assess possible association (*p* < 0.20) between these factors, exposure, and outcome. We used multivariate logistic regression analyses to estimate the odds ratios (ORs) for having skin lesions when exposed to different levels of As (lifetime average or cumulative amounts × years) for the two sexes and for groups exposed already at < 1 year of age and ≥ 1 year of age. Potential confounding factors that changed the effect estimates in the multivariate regression model by > 5%, were considered confounders and were included in the model. The model fit was evaluated by *r*^2^, and precision in the estimates was expressed by 95% confidence intervals (CIs). Statistical analyses were performed using SPSS software (version 12.0.1; SPSS Inc., Chicago, IL, USA).

## Results

The field staff interviewed and examined 166,934 individuals (Figure 1), and 504 cases with skin lesions were confirmed by experts and participated in the study. In total, 1,830 referents (94% of the eligible individuals) participated (Figure 1). In a comparison of participating and nonparticipating referents, nonparticipating referents were older and had higher education than those participating (Table 1). However, the current As exposure, evaluated at the household level because non-participating referents could not be interviewed, was not different in the nonparticipating referents compared to those participating (66% and 67% had drinking water > 50 μg/L; *p* = 0.8). The referents were shown to be representative of the population with respect to age, sex, education, and asset score (Table 1). The As exposure among the referents was slightly higher than the population as a whole (73 and 68% of the referents and the study population, respectively, had an average exposure of > 50 μg/L; *p* < 0.01).

Table 2 shows the distribution of cases and referents by age, sex, education, and SES. Cases and referents had different age distribution (40 vs. 30 years, on average), men dominated among cases (54 vs. 46%), cases more often had secondary or higher education (40 vs. 15%), and cases had higher household asset scores (58 vs. 41% with scores of 4–5) than referents.

The total number of wells ever used by participants was 6,174. Cases had significantly (*p* < 0.01) higher average chronic As exposure (since 1970) than referents (male cases, 200 μg/L; female cases, 211 μg/L; male referents, 143 μg/L; female referents, 155 μg/L) as well as higher cumulative exposure (male cases, 6,059 μg/L × years; female cases, 6,323 μg/L × years; male referents, 3,067 μg/L × years; female referents, 3,464 μg/L × years).

Cases were slightly older than referents when they first were exposed to As via the tube-well water (male cases, 13.6 years; female cases, 11.3 years; male referents, 10.4 years; female referents, 10.2 years, on average). Thus, male cases had similar average and cumulative exposure as female cases and started using tube-well water at about the same age. However, among the referents, women had higher exposure than men (*p* = 0.06 and *p* = 0.01 for average and cumulative exposure, respectively).

For each sex we found a significant dose–response relationship between average or cumulative As exposure and the risk of As-related skin lesions, using the lowest exposure category as reference (*p* < 0.001; Table 3). The effect estimates were similar when adjusting for age and asset score.

In the next step, we attempted to assess whether the risk of having skin lesions differed between females and males for the same As-exposure intervals. In a model with males and females combined, adjusted for age and asset score, males had a significantly higher risk of developing skin lesions than females, considering women’s lowest average exposure quintile as reference (Table 4). The difference in ORs between the sexes was especially prominent for the fifth average exposure quintile (*p* = 0.005). We saw the same pattern for cumulative exposure quintiles. The risk was significant at the fifth quintile (*p* < 0.001), and the adjusted risks were 5-fold higher for females (95% CI, 2.9–9.6) and 10-fold for men (95% CI, 6.1–19.8).

We also analyzed the risk of skin lesions by age at first exposure to tube-well water among those born after 1970 (up to 33 years of age at the time of screening; 146 cases, 977 referents). As shown in Table 5, subjects who had been exposed to drinking water from tube wells since before 1 year of age did not have a higher risk of developing skin lesions for the same quintiles of cumulative As exposure. On the contrary, for the lowest exposure quintile, those exposed before 1 year of age had a significantly lower OR than those who started using tube-well water later. The size of the data set did not allow for analysis of females and males separately.

## Discussion

This is the first large population-based epidemiologic study of the association between individual As exposure and skin lesions, and the first study designed to evaluate sex-specific dose–response patterns and possible effect modifications by age at first exposure. The use of referents randomly selected from the entire population enabled us to clarify that males had a higher risk of developing skin lesions than females, in line with the hypothesis. A start of exposure from before 1 year of age did not increase the risk of developing skin lesions for higher exposure quintiles. Unexpectedly, for lower exposure levels, the individuals exposed since or before birth had a lower risk of developing skin lesions than those exposed after 1 year of age.

The present study was based on screening of the entire population > 4 years of age (*n* = 166,934) for As-induced skin lesions. It included a two-step clinical process of evaluating skin lesions identified by well-trained community health workers in the field, because early or mild cases can easily be overlooked if the skin is not carefully investigated and compared with normal skin (Guha Mazumder et al. 1998; Rahman et al. 2003). The community health workers were instructed to include all suspected cases (maintaining high sensitivity); only about 34% of the skin lesions referred from the field were diagnosed as As-related skin lesions by the physicians, and 30% (504 cases) were ascertained by the dermatologists (Rahman et al. 2006). Thus, it is unlikely that cases with skin lesions were missed in the field. This is also supported by the fact that the physicians found no further skin lesions among the 1,830 referents, who had been classified as negative with regard to skin lesions by the field staff. Also, the field workers identified an expected number of six, later confirmed, cases within the listed group of randomly selected referents, corresponding to the prevalence found in the entire population. The referents, randomly selected from the population, had similar distributions of As exposure and covariates as in the entire study population. Nonparticipation was absent in the cases, and very few referents did not participate. Nonparticipating referents did not have different As concentrations in their household water sources.

Measurements of the water As concentrations were performed after the clinical skin examinations to avoid bias in the identification of As-related skin cases. To minimize information bias of historic well-water consumption, the information on drinking-water sources obtained in the interviews was instantly validated using data on source of drinking water from household surveys conducted in 1974, 1982, and 1996. Identification of the water source(s) used at each residence was complicated because calendar years are not widely observed in Bangladesh; therefore, years may be recalled inaccurately. To minimize such effects, we asked exposure histories in relation to momentous life events. Nonparticipating referents were older and had higher SES, which may have been linked to higher-than-average As exposure. However, nonparticipation was very limited, reducing the risk for selection bias.

We found an independent relationship between higher risk of skin lesions and higher assets and education. Higher SES and education groups took the lead in getting tube-well water in the 1970s and 1980s. This may explain why skin lesions were more common in those groups.

We found dose–response relationships with significant trends between average and cumulative exposures and skin lesions for both males and females. However, males had a higher risk of developing skin lesions than females in all categories (Table 3). Considering mean lifetime As exposure, males had twice the risk of obtaining skin lesions as females in the highest exposure quintile (Table 4). The mechanism behind this is not clear. An involvement of hormone interactions is possible, because As has been shown to interact with estrogen (Kitchin and Wallace 2005; Waalkes et al. 2004), which affects all the cell types of importance for skin physiology (e.g., epidermal keratinocytes, dermal fibroblasts, melanocytes) (Thornton 2005). In addition, differences between the sexes in the metabolism of As might have influenced the likelihood of developing skin lesions. Compared with females, males often have a higher fraction of the monomethylated As metabolite monomethylarsonate in urine (Hopenhayn-Rich et al. 1996; Vahter et al. 2002), which has been associated with increased risk of As-related skin lesions, including skin cancer (Chen et al. 2003; Del Razo et al. 1997). We are presently investigating potential differences in As metabolism between the sexes within the current study population.

Other susceptibility factors possibly involved in the observed differences in As-related skin lesions between males and females include water intake, sun exposure, smoking habits, and genetics. The higher risk of As-induced skin lesions in men than in women has been hypothesized to be caused by a higher intake of water (and thereby high As intake) because of higher physical activity. However, the males and females in the present cohort had similar urine As concentrations, which speaks against such an explanation. The urinary concentration of As metabolites (adjusted to a specific gravity of 1.015 g/ml) was 193 ± 240 μg/L (mean ± SD) in males and 209 ± 294 μg/L in females (Nermell B, Lindberg AL, Vahter M, personal communication). This analysis was based on the skin lesion cases (*n* = 504) and referents (*n* = 1,575) who provided urine samples at the clinical examination.

Generally, men are probably more exposed to the sun than women in Matlab, where rice cultivation and fishing are the most common occupations among men, and women are mainly occupied in domestic work. This may imply an increased risk of toxic effects in the skin, because interactions between As and ultraviolet (UV) irradiation for oxidative cell damage and cocarcinogenicity has been demonstrated in mouse skin (Uddin et al. 2005). Possibly, As-induced resistance to apoptosis in the skin may allow UV-damaged cells to escape normal cell population control (Pi et al. 2005). However, the role of sunlight is not all that obvious, because women had a prevalence of pigmentation changes similar to that of men (Rahman et al. 2006). Also, hyperkeratosis, which was more frequent among men, was not present on the most sun-exposed parts of the body, but rather on the palms of the hands and soles of the feet. Because about 70% of adult men in rural Bangladesh smoke, compared with < 1% of women, the role of smoking in the observed sex differences needs to be evaluated. Both As and smoking are potent inducers of oxidative stress (An et al. 2004; Helmersson et al. 2005; Nishigori et al. 2004; Pi et al. 2003), and a recent small-scale study suggested that genetic susceptibility to oxidative stress, as determined by polymorphisms in the myeloperoxidase and catalase genes, is associated with elevated risk of developing As-related hyperkeratosis (Ahsan et al. 2003). Also, As skin lesions in Inner Mongolia were shown to be related to markers of oxidative DNA damage (Fujino et al. 2005). However, no association was found between smoking and As-related skin cancer (Chen et al. 2003) or cutaneous squamous cell carcinoma (Odenbro et al. 2005), although overexpression of ras p21 oncoproteins has been suggested to play a role in the initiation and progression of oral squamous cell carcinoma in smokers (Kuo et al. 1995).

Contrary to the hypothesis, individuals exposed since birth, or before, did not show a higher risk of skin lesions than individuals who were exposed later in life. In fact, individuals who had been moderately exposed from or before birth were less prone to develop As-related skin lesions than those who were ≥ 1 year of age when they started using tube-well water. However, this was not because of lower life-time exposure; these individuals exposed earlier in life had slightly higher cumulative As exposure than those exposed later in life. Considering the transfer of As over the placenta (Concha et al. 1998a, 1998b), a lower risk estimate for individuals exposed since before birth is indeed an unexpected finding. Although we cannot rule out the possibility of induction of a protective mechanism, such as the thioredoxin-dependent embryonic acquisition of tolerance to oxidative stress, induced primarily by increased oxygen pressure after the utero–placental circulation is established (Kobayashi-Miura 2002), it is less likely. This mechanism would protect against other As-related effects as well. In contrast, our ongoing studies on the reproductive effects of As in Bangladesh show that As exposure during pregnancy increases the risk of fetal and, in particular, infant mortality (Rahman A, Vahter M, Ekstrom EC, Rahman M, Mustafa AHMG, Wahed MA, Yunus M, Persson LA, personal communication). Thus, As exposure early in life may eliminate the most susceptible individuals, thereby giving a wrong impression of a low risk for skin effects later in life. Because of increasing evidence that early-life exposures affecting fetal and infant growth (Moore et al. 2003; Yang et al. 2003) may cause chronic disease later in life (Lucas et al. 1999), more research concerning the health risks of early As exposure is highly warranted. Indeed, recent experimental studies showing hypersensitivity to As carcinogenesis in mice exposed to As only during gestation (Waalkes et al. 2004) support the need of human data in this respect.

Given the extent of As exposure in Bangladesh, a considerable proportion of the future disease burden may be attributed to As exposure; hence, public health interventions are urgently required. Currently, pond-sand filters, tube-well filters, and rainwater harvesting are being introduced to supply As-free drinking water to the exposed population (Jakariya et al 2005). These options are short-term measures. The long-term solution may include the provision of a piped water supply and the optimum use of surface water, which has been successfully used in other countries (e.g., Taiwan, Chile). The potential role that local governments can play in the long-term vision must be fully explored; toward this end, experimentation and pilot projects should not wait to mitigate this catastrophe. There may be several susceptibility factors that would justify prioritized mitigation activities. We are currently analyzing data to determine the association between As exposure and skin lesions in relation to nutritional status and As metabolism.

## Figures and Tables

**Table 1:** Distribution [no. (%)] of the study population and referents by age, sex, education, asset score, and average exposure to As in drinking water since 1970.

Variable	Study population (*n* = 166,934)	Referents (*n* = 1,830)	Referents vs. population^a^	Nonparticipant referents (*n* = 119)	Participating vs. nonparticipating referents^a^
Age (years)					
5–14	43,028 (26)	495 (27)		18 (15)	
15–24	35,923 (22)	382 (21)		44 (37)	
25–34	23,976 (14)	256 (14)		21 (18)	
35–44	24,367 (15)	280 (15)		17 (14)	
45–54	15,801 (9)	159 (9)		8 (7)	
> 55	23,839 (14)	258 (14)	0.63	11 (9)	< 0.01
Sex					
Male	74,408 (45)	833 (46)		56 (47)	
Female	92,526 (55)	997 (54)	0.42	63 (53)	0.8
Education					
None	89,243 (53)	985 (54)		49 (41)	
Primary	49,743 (30)	565 (31)		38 (32)	
Secondary	23,897 (14)	243 (13)		26 (22)	
Higher	4,050 (2)	37 (2)	0.34	6 (5)	< 0.01
Household asset score					
0 (missing)	19,761 (12)	188 (10)		22 (18)	
1 (very poor)	24,050 (14)	254 (14)		20 (17)	
2	29,263 (18)	316 (17)		21 (18)	
3	30,036 (18)	329 (18)		18 (15)	
4	33,761 (20)	412 (23)		22 (18)	
5 (rich)	30,063 (18)	331 (18)	0.11	16 (13)	0.06
Average historic As exposure					
< 50 μg/L	53,305 (32)	491 (27)			
> 50 μg/L	113,629 (68)	1,339 (73)	< 0.01		

a *p*-Value for chi-square test.

**Table 2:** Distribution [no. (%)] of interviewed cases and referents by age, sex, education, and asset score.

Variable	Cases (*n* = 504)	Referents (*n* = 1,830)	Cases vs. referents^a^
Age (years)			
5–14	2 (0)	495 (27)	
15–24	61 (12)	382 (21)	
25–34	106 (21)	256 (14)	
35–44	168 (33)	280 (15)	
45–54	94 (19)	159 (9)	
> 55	73 (14)	258 (14)	< 0.01
Sex			
Male	272 (54)	833 (46)	
Female	232 (46)	997 (54)	< 0.01
Education			
None	155 (31)	985 (54)	
Primary	148 (29)	565 (31)	
Secondary	141 (28)	243 (13)	
Higher	60 (12)	37 (2)	< 0.01
Household asset score			
0 (missing)	40 (8)	188 (10)	
1 (very poor)	48 (10)	254 (14)	
2	52 (10)	316 (17)	
3	73 (14)	329 (18)	
4	120 (24)	412 (23)	
5 (rich)	171 (34)	331 (18)	< 0.01

a *p*-Value for chi-square test.

**Table 3:** Distribution [no. (%)] of cases and referents by sex with regard to mean (μg/L) water As concentrations, cumulative As exposure (μg/L × years) since 1970, and crude and adjusted OR (95% CI).

Sex/exposure	Cases (*n* = 504)	Referents (*n* = 1,830)	Crude OR (95% CI)	Adjusted OR (95% CI)^a^
Mean As exposure (μg/L)				
Male				
< 10^b^	13 (4.8)	103 (12.4)	1.0	1.0
10–49	38 (14.0)	120 (14.4)	2.51 (1.27–4.97)	3.25 (1.43–7.38)
50–149	59 (21.7)	264 (31.7)	1.77 (0.93–3.37)	2.28 (1.04–4.98)
150–299	110 (40.4)	251 (30.1)	3.47 (1.87–6.45)	5.41 (2.52–1.62)
≥ 300	52 (19.1)	95 (11.4)	4.34 (2.22–8.46)	9.56 (4.20–21.8)
Total	272	833		
Female				
< 10^b^	12 (5.2)	127 (12.7)	1.0	1.0
10–49	15 (6.5)	141 (14.1)	1.13 (0.51–2.50)	1.66 (0.65–4.24)
50–149	65 (28.2)	287 (28.8)	2.40 (1.25–4.59)	3.06 (1.39–6.74)
150–299	84 (36.2)	300 (30.1)	2.96 (1.56–5.62)	4.08 (1.86–8.93)
≥ 300	56 (24.1)	142 (14.2)	4.17 (2.14–8.14)	6.88 (3.06–15.5)
Total	232	997		
Cumulative As exposure (μg/L × years)				
Male				
< 1,000^b^	37 (13.6)	213 (25.6)	1.0	1.0
1,000–4,999	75 (27.6)	453 (54.4)	0.95 (0.62–1.46)	1.05 (0.65–1.68)
5,000–9,999	119 (43.8)	143 (17.2)	4.79 (3.13–7.33)	4.50 (2.80–7.22)
> 10,000	41 (15.1)	24 (2.9)	9.83 (5.33–18.15)	10.4 (5.27–20.5)
Total	272	833		
Female				
< 1,000^b^	22 (9.5)	256 (25.7)	1.0	1.0
1,000–4,999	78 (33.6)	482 (48.4)	1.88 (1.15–3.09)	1.94 (1.10–3.42)
5,000–9,999	87 (37.5)	209 (25.1)	4.84 (2.93–8.00)	4.50 (2.54–7.99)
> 10,000	45 (19.4)	50 (5.0)	10.47 (5.79–18.95)	9.19 (4.77–17.7)
Total	232	997		

a Adjusted for age and asset score.

b Reference group; chi-square test for dose–response trend for unadjusted ORs, *p* < 0.001 (all categories).

**Table 4:** Mean lifetime As exposure (quintiles of distribution, μg/L) and risk of As-related skin lesions (hyperkeratosis and/or pigmentation changes) by sex.

Mean As exposure quintiles	Sex	Mean As exposure	Adjusted OR (95% CI)^a^
1	Female	8.3	1.0^b^
	Male	9.8	2.29 (1.16–4.53)
2	Female	60.0	1.22 (0.60–2.48)
	Male	59.3	1.99 (1.03–3.87)
3	Female	124	2.64 (1.40–4.00)
	Male	127	2.67 (1.37–5.20)
4	Female	199	2.87 (1.52–5.41)
	Male	199	3.88 (2.04–7.37)
5	Female	370	5.78 (3.10–10.8)^c^
	Male	344	10.9 (5.80–20.4)^c^

Results adjusted for age and household asset scores. Model: *r*^2^ = 0.32; cases, *n* = 464; referents, *n* = 1,639.

a Adjusted for age and asset score.

b Reference group.

c Difference between sexes within the fifth quintile; *p* = 0.005.

**Table 5:** Cumulative As exposure, by quintiles of distribution, and risk of As-related skin lesions (hyperkeratosis and/or pigmentation changes) for individuals exposed before 1 year of age and at ≥ 1 year of age.

Cumulative As exposure quintiles	Age at first exposure to tube-well water (years)	Mean As exposure (years × μg/L)	Adjusted OR (95% CI)^a^
1–2	≥ 1	596	1.0^b^
	< 1	830	0.27 (0.09–0.87)
3–4	≥ 1	3,188	1.10 (0.52–2.32)
	< 1	3,302	0.98 (0.50–1.94)
5	≥ 1	7,904	3.73 (1.75–7.98)^c^
	< 1	8,109	3.34 (1.79–6.24)^c^

Individuals born 1970 or later are included in the analysis. Cases, *n* = 146; referents, *n* = 977. Results were adjusted for age, sex, and household asset score. Model: *r*^2^ = 0.34.

a Adjusted for age, sex, and asset score.

b Reference group.

c Difference between groups exposed to tube-well water from < 1 year of age and ≥ 1 year of age within fifth quintile; *p* = 0.75.
